# Cybervictimization of Adults With Long-term Conditions: Cross-sectional Study

**DOI:** 10.2196/39933

**Published:** 2023-05-17

**Authors:** Zhraa A Alhaboby, James Barnes, Hala Evans, Emma Short

**Affiliations:** 1 The Open University Milton Keynes United Kingdom; 2 Fatima College of Health Sciences Abu Dhabi United Arab Emirates; 3 Coventry University Coventry United Kingdom; 4 London Metropolitan University London United Kingdom

**Keywords:** cyberharassment, chronic conditions, disability, social media, cyberbullying, web-based hate

## Abstract

**Background:**

People living with chronic conditions and disabilities experience harassment both offline and on the web. Cybervictimization is an umbrella term for negative web-based experiences. It has distressing consequences on physical health, mental well-being, and social relationships. These experiences have mostly been documented among children and adolescents. However, the scope of such experiences is not well documented among adults with long-term conditions, and the potential impact has not been examined from a public health perspective.

**Objective:**

This study aimed to examine the scope of cybervictimization among adults living with long-term conditions in the United Kingdom and the perceived impact on self-management of chronic conditions.

**Methods:**

This paper reports the findings of the quantitative phase of a mixed methods study in the United Kingdom. This cross-sectional study targeted adults aged ≥18 years with long-term conditions. Using a web-based link, the survey was shared on the web via 55 victim support groups, health support organizations, and social media accounts of nongovernmental organizations and activists such as journalists and disability campaigners. People with long-term conditions were asked about their health conditions, comorbidities, self-management, negative web-based experiences, their impact on them, and support sought to mitigate the experiences. The perceived impact of cybervictimization was measured using a set of questions on a Likert scale, frequency tables, and the Stanford Self-Efficacy for Managing Chronic Diseases Scale. Demographic data and the impact on self-management were cross-tabulated to identify the demographic characteristics of the targeted individuals and potential conditions with complications and highlight directions for future research.

**Results:**

Data from 152 participants showed that almost 1 in every 2 adults with chronic conditions was cybervictimized (69/152, 45.4%). Most victims (53/69, 77%) had disabilities; the relationship between cybervictimization and disability was statistically significant (*P*=.03). The most common means of contacting the victims was Facebook (43/68, 63%), followed by personal email or SMS text messaging, each accounting for 40% (27/68). Some participants (9/68, 13%) were victimized in web-based health forums. Furthermore, 61% (33/54) of victims reported that experiencing cybervictimization had affected their health condition self-management plan. The highest impact was on lifestyle changes such as exercise, diet, avoiding triggers, and avoiding excessive smoking and alcohol consumption. This was followed by changes to medications and follow-ups with health care professionals. Most victims (38/55, 69%) perceived a worsened self-efficacy on the Self-Efficacy for Managing Chronic Diseases Scale. Formal support was generally rated as poor, with only 25% (13/53) of victims having disclosed this experience to their physicians.

**Conclusions:**

Cybervictimization of people with chronic conditions is a public health issue with worrying consequences. This triggered considerable fear and negatively influenced the self-management of different health conditions. Further context- and condition-specific research is needed. Global collaborations to address inconsistencies in research are recommended.

## Introduction

### Background

Millions of people worldwide live with chronic health conditions, and the prevalence of such conditions is projected to increase [1]. The term *chronic* is derived from the Greek word *khronos*, which means *time*, and the dictionary definition for a chronic condition is an illness that persists for a long time or with a recurring nature [2]. In medicine, chronicity covers a group of diseases characterized by recurrence and slow progression. The medical definition of chronicity includes communicable conditions resulting from infectious agents such as tuberculosis. In public health and through the lens of international health organizations, *chronic disease* typically refers to noncommunicable diseases, which are characterized by a duration of a year or longer with slow progression and required management that includes medical follow-up and lifestyle changes with or without pharmacological treatment [3]. Examples include cardiovascular diseases, diabetes, cancers, and chronic respiratory diseases such as asthma and chronic obstructive pulmonary disease [1,4]. These represent the leading causes of morbidity and mortality worldwide [1]. The public health definition of chronic disease is the one adopted in this study.

Chronic conditions and disabilities overlap in terms of definition and day-to-day experiences. Hence, a chronic disease can result in disability and vice versa [5]. For example, 25% of people with chronic conditions have disabilities, and 80% to 90% of people with disabilities have chronic conditions [6]. The Equality Act 2010 in the United Kingdom defines disability as a “physical or mental impairment and the impairment has a substantial and long-term adverse effect on [an individual’s] ability to carry out normal day-to-day activities” [7]. A total of 14.6 million people in the United Kingdom had a disability in the year 2020 to 2021, which represents 22% of the total population [8]. It is important to note that not all impairments are chronic conditions and not all chronic conditions are disabling; however, they overlap considerably. The major points in this research are the chronicity factor, which indicates that a person is living with a condition, and the self-management aspect, which reflects the day-to-day changes to lifestyle or medications to manage the condition. To reflect this, from this point onward, the conditions covered in this paper are referred to as *long-term conditions* or *chronic conditions*. Disability will be specifically highlighted in questions pertaining to disability.

Living with a long-term condition is physically and mentally demanding to manage on a daily basis. This is further complicated by being treated differently in society. The *offline* targeting of people with long-term conditions is a documented phenomenon among young individuals [9] and has also been reported as hate incidents against adults with disabilities [10]. The increase in web-based communication has further reshaped this phenomenon to include *online targeting*, or cybervictimization.

A systematic review examined the experiences and impact of cybervictimization of people with long-term conditions and disabilities [11]. The narrative synthesis of the reported results covered a total of 3070 people with chronic conditions from 10 included studies. The sample sizes ranged from 42 to 823 participants, and the age range was 6 to 71 years. The reported prevalence range of cybervictimization was 2% to 41.7% [12]. The risk of being targeted was consistent for people with long-term conditions, who were described as being “different.” Such differences might include visible physical differences; invisible neurodiversity; or differences in lifestyle management of the health condition, such as using an inhaler or insulin pump in front of peers [13-15]. However, researchers from different disciplines and countries have used various terminologies to address such web-based incidents.

The terminology related to the negative web-based experiences of people with long-term conditions included cyberbullying, cyberstalking, cyberharassment, cyberhate, and cybervictimization. *Cyberbullying* is a term used to describe web-based abuse that involves a power imbalance between the victim and offender; it was the most commonly used term in previous studies [13]. Owing to its emphasis on perceived differences in power, cyberbullying is a term used with young victims, such as in schools, or workplaces where the victim has less authority than the perpetrator [11]. Cyberstalking is another term used [16], which is characterized by fixation and persistence. Such persistence can also be seen in cases of cyberhate and disability hate crimes in which victims experienced repetitive harassment from similar groups with a fixation on the impairment [17]. Cybervictimization and cyberharassment were used as generic terms to describe the experience of intimidation or abuse using web-based communication [14,16]. Accordingly, because of such differences among researchers and to facilitate communication internationally, the umbrella term *cybervictimization* was adopted in this research.

The reported scope and impact of cybervictimization lacks examination of the phenomenon in older age groups. Moreover, limited studies have focused on health consequences. In a cross-sectional study in Sweden [14], a sample of 8544 individuals was examined, of whom 762 had disabilities, aged 12, 15, and 17 years. The impact on the victims was mainly subjective health complaints [14]. Another public health study in Sweden [13] looked at 413 participants aged 13 to 15 years. The reported impact of web-based experiences included poor health, mental health consequences, and self-harm. Both studies [13,14] provided insights into the impact of cybervictimization on health; however, the target population was not adults.

In the United Kingdom, individuals with long-term conditions comprise 30% of the population, 64% of outpatient appointments, and 70% of inpatients [18].

### Objectives

No previous research has examined the web-based experiences of people with long-term conditions in the United Kingdom [11]. A relatively recent petition was raised to the House of Commons in the United Kingdom with concerns about the cybervictimization of people with disabilities. This was followed by investigations, and the governmental report acknowledged the concerns over the cyberabuse of people with long-term conditions and disabilities. It recommended further legislative and nonlegislative acts to prevent such experiences and their long-term impact on health [19]. The research reported in this paper was used to inform this governmental report to identify the impact of cybervictimization on people with long-term conditions. This study aimed to examine the scope and impact of cybervictimization of people with long-term conditions in the United Kingdom.

## Methods

### Ethics Approval and Risk Assessment

Ethics approval was granted by the University Research Ethics Committee at the University of Bedfordshire, United Kingdom (IHRREC C557). Ethical considerations were an ongoing process owing to the sensitivity of the topic, which also included developing a risk assessment for participants and researchers. The risk assessment included categorizing the potential risks arising during the study from low to high; their likelihood; and what was planned to mitigate the risk, such as signposting to support channels, additional discussions with the ethics committee, or a need for disclosure to protect the participants from immediate harm.

### Target Population

The target population in this survey included individuals aged ≥18 years, of any gender, of any ethnic background, with a self-reported chronic condition or impairment of a minimum duration of 3 months, residing in the United Kingdom during the research period, and with internet access. Participants were identified as having a long-term condition if they responded “yes” to the following question: “Do you have a long-standing medical condition/illness or disability that requires monitoring, lifestyle changes, and/or taking medications? By long-standing, we mean anything that has affected you over a period of at least 3 months or that is likely to affect you over a period of at least 12 months.” To ensure that only eligible participants could complete the survey, a prescreening at the beginning of the survey confirmed the eligibility criteria. Any missing criterion was designed to lead to a “thank you” note and ending the survey.

### Survey Design

The survey questions were developed based on a literature review and discussions with experts in cyberharassment and further refined after the piloting stage. The final survey was put on the web on the Qualtrics website (Qualtrics International Inc) using an institutional account. This platform provided sufficient accessibility options for this research. The process of designing the questionnaire on the web included several tests to check the layout, question designs, and navigation between sections. A further check was conducted to ensure that the results reports reflected the main statistical output expected from each question. When the survey was fully functional, it was used for the piloting stage.

### Piloting

The development of tools included a pilot study conducted by the researchers over 4 weeks after obtaining ethics approval and before commencing the main data collection campaign. The aim of this stage was to test the functionality, clarity, and usability of the web-based questionnaire and obtain input from respondents on the wording or other areas of concern. The respondents were approached on the university campus and via direct contact with health care professionals. The researchers explained to the respondents that the study was a pilot test and invited them to fill out the questionnaire using a “think aloud” approach. The researcher asked the respondents to think loudly while completing the survey to obtain their real-time feedback on survey questions or use, which helped minimize memorization issues [20,21]. After completing the questionnaire, a short interview was conducted with a predesigned set of questions derived from the literature [22,23]. The set of questions covered the following points: (1) thoughts on the time to complete the questionnaire, (2) issues regarding the clarity of instruction, (3) overall layout, (4) confusing questions, (5) objectionable questions, and (6) additional comments to improve the survey.

There were 10 respondents representing various demographics in terms of age, gender, ethnicity, and occupation. In total, 40% (4/10) of them reported living with a long-term condition, and 20% (2/10) of them had gone through the experience of cybervictimization and provided answers and feedback based on their lived experiences. Respondents who did not have a long-term condition were given the chance to make several attempts at the questionnaire and provide different answers to give feedback on the clarity of the questions and layout. The approximate time spent filling out the questionnaire was approximately 15 minutes if all sections were answered. The piloting stage influenced the recruitment stage by adding prescreening questions. This resulted in moving 1 question to the prescreening to include only participants with long-term conditions. There were minor issues in skip logic that required technical support from the Qualtrics team. This stage also included changes to the wording and options in 6 questions (religion, health condition, level of fear and distress, clarification of web-based harassment, and options of contact by the harassers in 2 questions). The question on the self-efficacy for managing chronic conditions scale was understood by the respondents, and the results were in line with the expected statistics.

### Survey Sections

The survey was open to all visitors to the web page and did not require registration to the website. The survey page started with a prescreening to confirm 3 main criteria related to age, living in the United Kingdom, and having a long-term condition. This was followed by a briefing consent form. To fill out the questionnaire, participants had to confirm by ticking boxes that they understood the information given, the anonymity, the right to withdraw, and contact details for further information or to complain. The survey was voluntary, and the participants could skip questions, as highlighted in the consent form, to avoid eliciting distress. In addition, most questions included “not applicable” or “rather no say” as answer choices. The participants were also provided with a back button to check or change their answers if needed. A survey logic was implemented to show the participants the options they had selected in the previous questions or automatically skip questions not relevant to them. The questions followed this logic without the randomization of the question. The survey included validation questions to prompt giving a response without forcing it.

The survey had 6 major sections, each of which included a number of questions. To ensure accessibility, short questions were grouped into 1 page, and long questions that included matrix buttons or scales were placed on separate pages. The first section focused on demographic information, such as gender, ethnicity, employment, and county of residence. The main outcomes anticipated from this section were the sample description and victim characteristics. The second section focused on the long-term condition and self-management plan. The participants had to tick their conditions and duration and were given additional space to add any condition. The plan was to further group the written conditions during analysis according to the nearest medical diagnosis in the International Classification of Diseases, 10th Revision (ICD-10) for 2015 [24]. Participants with comorbidities were asked about the health condition that affected them the most.

The third section was about cybervictimization experience; it started with 2 questions to identify victims. The first question provided the definition of cybervictimization in this study and asked participants if they had experienced this. Cybervictimization in this research was defined as “unwanted repeated contact *via* the internet such as email, chartroom, online forum, social network, mobile phone message, or other electronic means that was used to harass, insult, embarrass, or spread lies about the victim.” The second question was a direct question about whether they considered themselves victims of web-based harassment. Fear associated with distress was also included in the third section of the survey as it has been documented that the psychological effects of victimization have more impact on health [25,26].

The fourth section explored the participants’ coping, self-management during or after the cybervictimization experience, and the perceived motivation for harassment [9,27,28]. The impact of cybervictimization on the self-management plan was examined in multiple questions using impact statements, a Likert scale, and a self-efficacy scale.

The fifth section was designed to examine the actions taken by the victims and the support received in response to their experience of cybervictimization. The last page invited participants to volunteer for the second qualitative phase, which will be reported elsewhere (Alhaboby et al, unpublished data, 2022).

### Using a Standardized Scale

Self-efficacy is a core concept in the self-management of chronic conditions; it represents patients’ own beliefs about how capable they are of taking control of managing their health conditions [29]. Hence, the Stanford standardized efficacy scale was used to examine the perceived impact on the self-management of health [30,31]. It comprises 6 questions to be answered with a score from 0 to 10, with the average of the 6 scores representing the self-efficacy of the participant [30].

The researchers aimed to examine the difference in self-efficacy in the self-management of chronic conditions before and after the experience of cybervictimization. The participants were asked to respond to the set of questions twice: once considering their self-management before cybervictimization and the second time considering the current self-management plan. A negative change before and after victimization could indicate a perceived disruption of the self-management plan [32]. The limitations of using the scale are discussed in the Strengths and Limitations section.

### Recruitment

Web-based recruitment was through victim support groups, patient support groups, and social media accounts of organizations and activists in the fields of cyberabuse or disability campaigners. Search engines were used to look for victim and health support groups. The keywords used included *patient*, *support*, *chronic*, *health forum*, *disability*, *hate crime*, *online support*, and specific health condition names. The inclusion criteria for gatekeepers were (1) established patient and victim support group or organization, (2) based in the United Kingdom or with a considerable audience from the United Kingdom, (3) having terms and policies on their websites aligning with ethics to protect participants [33], (4) having direct contact with patients or victims, and (5) providing contact details. Further snowballing was conducted to reach relevant organizations, charities, journalists, academics, and activists in the field. The lead researcher contacted *gatekeepers* via email. When no response was received within 1 to 2 weeks, an email reminder was sent. In cases where a telephone number was provided, further contact via phone was made. Gatekeepers were provided with information related to the rationale of the study, expected benefits to participants in the short and long term, inclusion criteria, the survey link, the study poster, and contact details. Gatekeepers who agreed to collaborate in this research and help in recruitment sent the survey link to potential participants via their mailing lists, social media accounts, and monthly updates.

The recruitment process uncovered challenges in reaching the target population because of the sensitivity of the topic, especially as a considerable number of victims were still experiencing harassment. In total, 4 overarching themes influenced the recruitment process: social identity in online support groups, the influencing role of web-based gatekeepers, the contradictory role of social media, and the promotion of inclusivity. The challenges and lessons learned from web-based recruitment on this sensitive topic were theorized using social identity theory and published elsewhere [34].

### Data Collection Process

The average time to finish the survey was 15 minutes; it was longer for participants who completed sections relevant to cybervictimization. This was consistent between the pilot and main studies. There were daily checks of responses by the researchers to screen IP addresses, filter bots, and remove duplicate responses or false victimization cases. A separate screening form was developed by the research team for cases of suspected false victimization. False victimization refers to responses that raise suspicions of being factitious or associated with delusional disorders. The screening tool was used only once in this study, and the suspicious response was excluded from the analysis.

The data were anonymized with no means to be traced to the participants’ identity and stored in accordance with the Data Protection Act 1998. Anonymized data were stored in a password-protected device, and the data were shared only for analysis with the research team. The data set was not put in an open repository because of the sensitivity of the topic and as another level of reassurance for participants.

### Analysis

The survey data were collected over 18 months, from September 2015 to the end of March 2017. Incomplete responses were recorded 48 hours after the participants’ last activity. A total of 424 individuals accessed the survey on the web; 310 (73.1%) of them were eligible based on the prescreening, with 222 (52.4%) consenting to participate and 152 (35.8%) completing >50% of the survey. This was the final number included in the analysis.

The first step in the analysis was to use univariate statistics for descriptive statistics [35]. The participants reported various chronic conditions or disabilities. The demographic data were presented, followed by information on the long-term conditions. To ensure consistency and accuracy in categorizing and reporting these conditions, each response was categorized in accordance with the ICD-10 [24,36]. Owing to variations in terminology used by the participants, each condition entry was checked manually and cross-checked individually with the ICD-10.

The prevalence of cybervictimization in the sample was calculated, and descriptive statistics of the victimization experience were presented. Fear or distress was presented on a Likert scale and also grouped into a binary outcome as fear versus no fear [25]. The number of respondents in this section was variable to allow for skipping questions with which they were not comfortable. Hence, the frequency reflects the number of respondents to a specific question.

The impact of cybervictimization was analyzed using descriptive statistics and the calculation of the self-management efficacy scale. For each participant, the scale was calculated before and after or during victimization, as described previously.

The third step in analyzing the survey data was to cross-tabulate among the independent variables. Cross-tabulation was used to identify different factors in relation to the scope and impact of cybervictimization. Statistical significance tests were performed using Stata (version 12; StataCorp). The main independent variables were gender, ethnicity, age, disability status, and impact of cybervictimization. Statistical significance was measured using the chi-square test to examine the observed versus expected number of 2 × 2 tables, with a *P* value of significance of <.05. The Fisher exact test was used when the number in any cell was <5 [35]. To examine victims’ characteristics, cross-tabulations were made to highlight the main characteristics of victims with disabilities and compare them with those of the entire sample.

## Results

### Demographics

The sample (N=152) was diverse in terms of gender, ethnic background, and age. Of the 152 participants, 120 (78.9%) were female, 29 (19.1%) were male, and 3 (2%) did not specify their gender. The sample included 86.2% (131/152) of respondents from White ethnic backgrounds, 7.2% (11/152) of respondents of Asian ethnicity, 2.6% (4/152) of respondents from a mixed background, 2% (3/152) of respondents from a Black ethnic background, and 2% (3/152) of respondents from other or Arab background. The age range of the participants was 18 to 65 years, with a mean age of 34.74 (SD 12.98) years, and most were aged between 18 and 29 years (66/152, 43.4%). However, the age distribution included participants from different age groups: 21.7% (33/152) of the participants were aged 30 to 39 years, and 16.4% (25/152) of the participants were aged ≥50 years. A total of 67.1% (102/152) of the participants considered themselves disabled. At the time of data collection, 84.2% (128/152) of the participants were living in England across 42 counties. The sample also included participants from other parts of the United Kingdom: 6.6% (10/152) lived in Wales, 5.9% (9/152) lived in Scotland, and 3.3% (5/152) lived in Northern Ireland.

The respondents were asked about their employment status. Some participants in this question chose multiple options, and others skipped it. On the basis of the categories provided in the national guidance [37], the employment status of participants varied: 27.6% (42/152) were employed full time, 27.6% (42/152) were students, 14.5% (22/152) were unemployed, 7.9% (12/152) were employed part time, 7.2% (11/152) were self-employed, and 7.2% (11/152) were retired.

### The Diversity of the Reported Long-term Conditions

The participants (N=152) had a wide range of health conditions, with most having multiple comorbidities. Hence, 340 health conditions and comorbidities were collectively reported. Chronic lower respiratory diseases were reported by 34.9% (53/152) of the participants. The second category was endocrine and metabolic diseases, which were reported by 30.3% (46/152) of the participants and included conditions such as diabetes mellitus, thyroid diseases, and Wilson disease. Mental and behavioral disorders were reported by 30.3% (46/152) of the participants in the sample: 2.6% (4/152) of the participants were living with autism spectrum disorder, and 2% (3/152) of the participants reported Asperger syndrome. Diseases of the skin—eczema and psoriasis—affected 26.3% (40/152) of the participants. A wide spectrum of nervous system diseases such as epilepsy was reported by 25% (38/152) of the participants. Diseases of the musculoskeletal system, such as rheumatoid arthritis and fibromyalgia, were reported by 23.7% (36/152) of the respondents. This category also included a range of connective tissue disorders such as hypermobility syndrome, gout, and scoliosis. Diseases of the digestive system, such as noninfective inflammatory bowel diseases, were reported by 15.8% (24/152) of the respondents. Other less common but no less debilitating conditions were reported, such as genitourinary conditions (15/152, 9.9%), circulatory system disorders (13/152, 8.6%), congenital malformations or chromosomal abnormalities (10/152, 6.6%), neoplasms (9/152, 5.9%), hearing impairments (4/152, 2.6%), visual impairments (3/152, 2%), and injuries (3/152, 2%).

### The Experience of Living With a Long-term Condition

The participants (N=152) were asked about the condition that affected them the most. The top conditions were diabetes mellitus (23/152, 15.1%), psoriasis (14/152, 9.2%), Ehlers-Danlos syndrome (EDS; 10/152, 6.6%), myalgic encephalomyelitis (ME; 7/152, 4.6%), anxiety (7/152, 4.6%), depression (7/152, 4.6%), asthma (6/152, 3.9%), fibromyalgia (6/152, 3.9%), inflammatory bowel disease (6/152, 3.9%), multiple sclerosis (MS; 5/152, 3.3%), epilepsy (4/152, 2.6%), eczema (4/152, 2.6%), thyroid disease (3/152, 2%), Asperger syndrome (3/152, 2%), hypermobility syndrome (3/152, 2%), and renal disease (3/152, 2%).

Most participants (136/152, 89.5%) had been diagnosed with one or more long-term conditions by a physician in the United Kingdom.

The management plan of most participants involved multiple aspects; hence, 152 participants shared a total of 999 endorsements of elements of their health management plans. The most common element of health management was related to lifestyle changes, including avoiding triggers that exacerbate illness (93/152, 61.2%), healthy eating (77/152, 50.7%), avoiding excessive drinking (66/152, 43.4%), and physical activity (63/152, 41.4%). Pharmacological treatment was also reported by most participants, including regular (101/152, 66.4%) and prescription (76/152, 50%) medications.

### Cybervictimization Experience

Cybervictimization was found to be prevalent in this sample as 45.4% (69/152) of the participants were victimized on the web. The term “victim” will be used from this point onward to refer to this group for clarity. Owing to ethical considerations, responding to questions related to cybervictimization was voluntary; hence, the number of respondents in this section varies.

Among the victims (n=68), most reported experiencing fear and distress as a reaction to abusive communication (60/68, 88%), ranging from extreme fear and distress (22/68, 32%) to moderate fear (24/68, 35%) and slight fear (14/68, 21%).

The duration of the victimization was more than a year in 37% (25/68) of cases and between 3 months and 1 year in 22% (15/68) of cases. The harassment was ongoing in 25% (17/68) of cases, and 18% (12/68) of victims were not sure whether the harassment had ended.

The most common means of contacting the victims (n=68) included Facebook, as reported by 63% (43/68) of the victims, followed by personal email or SMS text messaging, each accounting for 40% (27/68) of victims, as detailed in Table 1. Phone calls were reported by 38% (26/68) of victims. Other means of contact included websites such as eBay, chat rooms, spam subscriptions, and hacking into friends’ accounts. Some participants (9/68, 13%) were victimized in web-based health forums. Most victims (67/68, 99%) were contacted once or more per day by their harassers.

Of the 68 victims, 20 (29%) reported that the harassers were strangers, 14 (21%) identified the harassers as acquaintances, and 9 (13%) reported that the harassers were ex-partners; however, 10 (15%) were unsure about the identity of their harassers. In addition, 24% (16/68) of victims specified other categories, such as neighbors, ex-partners’ partners, or fellow members of online support groups.

When the victims (n=53) were asked whether they considered having this chronic condition or impairment to be related to the experience of being harassed on the web, 42% (22/53) responded “yes.” These participants were provided with a space to explain their answers, which included experiences of disability discrimination, harassers pretending to have the same health condition to get closer to them, or the longer time spent on the web because of the impairment. This finding was also examined in the qualitative phase of the study (Alhaboby et al, unpublished data, 2022).

To find commonalities and differences among the whole sample, all victims, and victims with disabilities, the characteristics of each of these groups were cross-tabulated and summarized in Table 2. The table shows the minimal demographic differences among the sample, participants who experienced victimization, and participants with disabilities who experienced victimization.

**Table 1:** The means used to contact the victims, with frequency and duration (n=68).

Means of contact	Once or more per day, n (%)	More than 3 times per week, n (%)	Once per week, n (%)	Once per month, n (%)	Less than once a month, n (%)	Total, n (%)
Facebook	13 (19)	14 (21)	4 (6)	7 (10)	5 (7)	43 (63)
Personal email	9 (13)	7 (10)	4 (6)	4 (6)	3 (4)	27 (40)
SMS text messaging (such as WhatsApp)	11 (16)	6 (9)	1 (1)	6 (9)	3 (4)	27 (40)
Phone calls	6 (9)	6 (9)	5 (7)	4 (6)	5 (7)	26 (38)
Other	8 (12)	6 (9)	3 (4)	2 (3)	6 (9)	25 (37)
Twitter	9 (13)	2 (3)	4 (6)	3 (4)	2 (3)	20 (29)
Blogs	5 (7)	1 (1)	2 (3)	1 (1)	4 (6)	13 (19)
Web-based health forums	3 (4)	2 (3)	1 (1)	2 (3)	1 (1)	9 (13)
Work email	2 (3)	1 (1)	0 (0)	2 (3)	0 (0)	5 (7)
YouTube	0 (0)	0 (0)	1 (1)	2 (3)	1 (1)	4 (6)
Instagram	1 (1)	0 (0)	0 (0)	1 (1)	1 (1)	3 (4)
Total	67 (99)	45 (66)	25 (37)	34 (50)	31 (46)	68 (100)

**Table 2:** Comparison among the main characteristics of all participants, victims, and victims with disabilities (N=152).

Characteristic				All participants with chronic conditions		Victims (n=69)		Victims with disabilities (n=53)
**Demographics**								
	Gender (female), n (%)		120 (78.9)		56 (81.2)		43 (81.1)	
	Ethnic background (White), n (%)		131 (86.2)		61 (88.4)		48 (90.6)	
	Age (years), mean (SD; range)		34.74 (12.98; 18-65)		36.87 (12.65; 18-63)		37.96 (13.10; 18-63)	
	Religion (none), n (%)		74 (48.7)		38 (55.1)		8 (15.1)	
	**Employment status, n (%)**							
		Employed full time	42 (27.6)		18 (26.1)		10 (18.9)	
		Student	42 (27.6)		15 (21.7)		12 (22.6)	
		Unemployed	22 (14.5)		20 (29)		19 (35.8)	
		Employed part time	12 (7.9)		5 (7.2)		3 (5.7)	
		Self-employed	11 (7.2)		11 (15.9)		5 (9.4)	
		Retired	11 (7.2)		4 (5.8)		4 (7.5)	
	**Profession, n (%)**							
		Professional	41 (27)		17 (24.6)		12 (22.6)	
		Service or sales	12 (7.9)		7 (10.1)		4 (7.5)	
		Clerical support	9 (5.9)		1 (1.4)		1 (1.9)	
		Manager	8 (5.3)		5 (7.2)		2 (3.8)	
		Technician or associate professional	6 (3.9)		1 (1.4)		1 (1.9)	
	**Sexual orientation, n (%)**							
		Straight	113 (74.3)		51 (73.9)		39 (73.6)	
		Gay or lesbian	10 (6.6)		5 (7.2)		4 (7.5)	
		Bisexual	12 (7.9)		7 (10.1)		7 (13.2)	
		Other	8 (5.3)		3 (4.3)		1 (1.9)	
		Prefer not to say	9 (5.9)		3 (4.3)		2 (3.8)	
**Cybervictimization experience, n (%)**								
	Fear or distress		N/A^a^		56 (81.2)		46 (86.8)	
	**Means of contact**							
		Facebook	N/A		43 (63.2)^b^		34 (70.8)^c^	
		Web-based health forums	N/A		9 (13.2)^b^		8 (16.7)^c^	
	Most common duration (more than a year)		N/A		25 (36.8)^b^		18 (37.5)^c^	
	Harasser identity		N/A		20 (29.4)^b^		16 (33.3)^c^	
	Perceived targeting because of health condition or impairment		N/A		22 (41.5)^d^		18 (48.6)^e^	

^a^N/A: not applicable.

^b^n=68.

^c^n=48.

^d^n=53.

^e^n=37.

### The Impact of Cybervictimization

Of 54 victims, most (n=33, 61%) reported that cybervictimization had resulted in an impact on their self-management of chronic conditions. Of these 33 participants, 32 (97%) provided more details, were shown customized management options generated from their earlier responses in the survey, and were asked to tick the parts of the health management plan that were affected. Most changes were under the lifestyle category, such as avoiding triggers that exacerbate illness (19/32, 59%) and healthy eating (12/32, 38%). They also included changes to medications, follow-up with general practitioners, and self-monitoring. A detailed breakdown of the affected aspects of the self-management plan is shown in Table 3.

The impact of cybervictimization on the self-management plan was further examined by asking the victims to endorse impact statements that applied to them, which were ranked on a 5-point Likert scale ranging from *always* to *never*. A total of 32 victims responded to this question, and their responses reflected multilevel effects on health management and provided potential explanations for the changes stated in Table 3. A detailed breakdown of the impact statements and their endorsements is reported in Table 4.

To identify the conditions that were more commonly victimized, these were cross-tabulated with cybervictimization. Owing to the low number, a statistical significance test was not performed, but highlighting these conditions is important for future research. These were mainly people with asthma, diabetes, depression, chronic obstructive pulmonary disease, anxiety, MS, ME, fibromyalgia, EDS, heart disease, thyroid disease, and inflammatory bowel disease.

The aforementioned reported results were further cross-checked to identify the impact of cybervictimization on each chronic condition reported in the sample, and this impact was shared with the UK government to guide future mitigating actions [19,38]. Table 5 summarizes the impact reported based on chronic conditions.

An additional step to measure the impact of cybervictimization included using the Stanford Self-Efficacy for Managing Chronic Diseases 6-item scale. The score was calculated for each victim (n=55) before and after the cybervictimization experience; it was negative in 69% (38/55) of responses, positive in 13% (7/55) of cases, and zero in 18% (10/55) of cases. Hence, a negative difference in scale indicates a perceived change in self-efficacy before and after the cybervictimization experience and potentially reflects a negative impact of cybervictimization on the self-management of chronic conditions.

The relationship between gender and being cybervictimized was not statistically significant, with a *P* value of .61 using the chi-square test. The Fisher exact test was used to examine the relationship between gender and the perceived impact on self-management; however, the result was a *P* value >.99, which was not statistically significant.

There was a statistically significant relationship between being a person with a disability and cybervictimization, with a *P* value of .03. However, there was no difference in the perceived impact of cybervictimization between victims with and without disabilities. The *P* value using the chi-square test was .19, which was not significant at *P*<.05.

Sexual orientation and employment status in relation to cybervictimization were not statistically significant. Reporting fear and distress was statistically significant with regard to the impact of cybervictimization (*P*<.001), as shown in Table 6.

The impact of the duration of cybervictimization was also examined. The chi-square test was not statistically significant, with a *P* value of .20. However, when the categories were narrowed to ≤1 year compared with >1 year, there was a significant relationship between the duration of cybervictimization and its perceived impact. The chi-square statistic was 4.8. The *P* value was .03, which was significant at *P*<.05.

**Table 3:** Victims’ responses to what specific aspects of the self-management of chronic conditions were affected after cybervictimization (n=32).

Affected aspects of the self-management of chronic conditions		Victims, n (%)
**Lifestyle changes (n=60 endorsements)**		
	Avoiding particular triggers that exacerbate illness	19 (59)
	Healthy eating	12 (38)
	Avoiding excessive drinking	5 (16)
	Exercise and physical activity	10 (31)
	Avoiding smoking	4 (12)
	Avoiding particular types of food	4 (12)
	Other lifestyle changes	6 (19)
**Pharmacological aspects (n=16 endorsements)**		
	Regular medications	9 (28)
	Medications on need (prescription)	4 (12)
	Medications on need (over the counter)	3 (9)
**Follow-up (n=14 endorsements)**		
	Regular follow-up with a specialist	2 (6)
	Regular follow-up with GP^a^	5 (16)
	Regular follow-up with other health care professionals	2 (6)
	Physiotherapy	0 (0)
	Counseling sessions	5 (16)
**Monitoring (n=5 endorsements)**		
	Self-monitoring at home (eg, blood sugar)	3 (9)
	Regular laboratory tests	2 (6)
**Other (n=3 endorsements)**		
	Alternative and complementary medicine (such as herbal treatment, aromatherapy, and acupuncture)	3 (9)
	Other management	0 (0)

^a^GP: general practitioner.

**Table 4:** The endorsements by victims of impact statements that applied to them on a 5-point Likert scale (n=32).

Statement	Always, n (%)	Most of the time, n (%)	Sometimes, n (%)	Rarely, n (%)	Never, n (%)
Being harassed made me ignore my medications.	2 (6)	6 (19)	10 (31)	6 (19)	8 (25)
I feel that my health never got back to how it was before being harassed.	11 (34)	6 (19)	7 (22)	2 (6)	6 (19)
Being harassed made me too tired to do exercise.	11 (34)	10 (31)	7 (22)	1 (3)	3 (9)
Being harassed made me too scared for outside exercise.	14 (44)	8 (25)	8 (25)	1 (3)	1 (3)
Being harassed affected my GP^a^ follow-up appointments.	3 (9)	5 (16)	10 (31)	5 (16)	9 (28)
Being harassed made me too scared to attend my appointments.	3 (9)	5 (16)	8 (25)	6 (19)	10 (31)
Being harassed affected my appetite and eating.	7 (22)	12 (38)	9 (28)	1 (3)	3 (9)
Being harassed affected my self-monitoring at home.	8 (25)	8 (25)	9 (28)	2 (6)	5 (16)
Being harassed made me take more medications than usual.	9 (28)	6 (19)	7 (22)	5 (16)	5 (16)
Being harassed made me take painkillers more than usual.	8 (25)	4 (12)	10 (31)	4 (12)	6 (19)
Being harassed made me take prescribed drugs.	7 (22)	7 (22)	4 (12)	6 (19)	8 (25)
Being harassed made me start smoking or smoking more than usual.	5 (16)	6 (19)	5 (16)	2 (6)	14 (44)
Being harassed made me start drinking alcohol or drinking alcohol excessively.	5 (16)	2 (6)	5 (16)	7 (22)	13 (41)
My treatment was the same but I felt worse after being harassed.	15 (47)	8 (25)	5 (16)	0 (0)	4 (12)
My treatment was the same but my lab tests deteriorated after being harassed.	4 (12)	3 (9)	8 (25)	5 (16)	12 (38)
After being harassed my treatment was the same but my physician says I am not doing well.	3 (9)	6 (19)	4 (12)	9 (28)	10 (31)
After being harassed my treatment was the same but my family or friends think I am not doing well.	6 (19)	10 (31)	9 (28)	2 (6)	5 (16)
Other effects	9 (28)	5 (16)	2 (6)	2 (6)	14 (44)

^a^GP: general practitioner.

**Table 5:** The impact of cybervictimization on the management plan of each reported condition.

Category and condition			Reported impact
**Endocrine, nutritional, and metabolic diseases**			
	Diabetes mellitus	Healthy eating (reported by multiple participants in this category) Avoiding particular triggers that exacerbate illness (reported by multiple participants in this category) Monitoring at home (eg, blood sugar) Avoiding particular types of food Avoiding smoking Avoiding excessive drinking Exercise and physical activity Regular medications	
**Mental and behavioral disorders**			
	GAD^a^	Other lifestyle changes such as relaxing Avoiding particular triggers that exacerbate illness Exercise and physical activity	
	Depression	Avoiding smoking Healthy eating (reported by multiple participants in this category) Regular follow-up with GP^b^ Counseling sessions Avoiding particular triggers that exacerbate illness Regular medications Alternative and complementary medicine (such as herbal treatment, aromatherapy, and acupuncture) Avoiding excessive drinking	
	Unspecified mental health condition	Healthy eating Counseling sessions Avoiding particular triggers that exacerbate illness Avoiding smoking Exercise and physical activity	
	Asperger syndrome	Avoiding particular triggers that exacerbate illness	
	PTSD^c^	Medications on need (over the counter) Regular follow-up with a specialist Regular follow-up with other health care professionals Counseling sessions Avoiding particular triggers that exacerbate illness Other lifestyle changes Regular medications	
	Bipolar affective disorder	Regular follow-up with other health care professionals Avoiding particular triggers that exacerbate illness Avoiding excessive drinking	
**Diseases of the nervous system**			
	ME^d^	Avoiding particular triggers that exacerbate illness Other lifestyle changes Avoiding excessive drinking	
	Epilepsy	Healthy eating Avoiding particular triggers that exacerbate illness Medications on need (prescription)	
	Migraine	Healthy eating	
	Narcolepsy	Healthy eating Self-monitoring at home (eg, blood sugar) Avoiding a particular type of food Avoiding particular triggers that exacerbate illness Avoiding excessive drinking Exercise and physical activity Regular medications	
	Restless leg syndrome	Regular follow-up with GP Avoiding particular triggers that exacerbate illness Regular medications	
**Diseases of the musculoskeletal system and connective tissue**			
	Rheumatoid arthritis	Healthy eating Medications on need (over the counter) Alternative and complementary medicine (such as herbal treatment, aromatherapy, and acupuncture) Other management Avoiding a particular type of food Avoiding particular triggers that exacerbate illness Exercise and physical activity Other lifestyle changes	
	Fibromyalgia	Healthy eating (reported by multiple participants in this category) Self-monitoring at home (eg, blood sugar) Other management Avoiding particular triggers that exacerbate illness Other lifestyle changes Regular medications (reported by multiple participants in this category) Medications on need (prescription) Regular follow-up with GP Avoiding a particular type of food Medications on need (prescription)	
**Diseases of the skin and subcutaneous tissue**			
	Eczema or acne	Healthy eating Alternative and complementary medicine (such as herbal treatment, aromatherapy, and acupuncture) Avoiding excessive drinking	
	Psoriasis	Exercise and physical activity (reported by multiple participants in this category)	
**Diseases of the genitourinary system**			
	Menstrual disorders	Healthy eating Regular follow-up with a specialist Counseling sessions Avoiding particular triggers that exacerbate illness Avoiding smoking Exercise and physical activity Regular medications	
**Diseases of the circulatory system**			
	Heart disease	Regular follow-up with GP (reported by multiple participants in this category) Avoiding particular triggers that exacerbate illness (reported by multiple participants in this category) Regular medications	
**Congenital malformations, deformations, and chromosomal abnormalities**			
	Ehlers-Danlos syndrome	Medications on need (over the counter) Medications on need (prescription) Alternative and complementary medicine (such as herbal treatment, aromatherapy, and acupuncture) Exercise and physical activity (reported by multiple participants in this category) Counseling sessions Avoiding particular triggers that exacerbate illness (reported by multiple participants in this category) Other lifestyle changes	

^a^GAD: generalized anxiety disorder.

^b^GP: general practitioner.

^c^PTSD: posttraumatic stress disorder.

^d^ME: myalgic encephalomyelitis.

**Table 6:** The relationship between the perceived impact of cybervictimization and fear or distress is statistically significant (n=54).^a^

Fear or distress	Victims who reported cybervictimization impact on self-management, n (%)	Victims who reported no cybervictimization impact on self-management, n (%)	Total, n (%)
Extreme fear or distress	16 (30)	1 (2)	17 (31)
Moderate fear or distress	12 (22)	6 (11)	18 (33)
Slight fear or distress	4 (7)	7 (13)	11 (20)
No fear or distress	1 (2)	7 (13)	8 (15)
Total	33 (61)	21 (39)	54 (100)

^a^The chi-square statistic was 18.8. The *P* value was <.001. This result was significant at *P*<.05.

### Support

The participants sought formal and informal support to cope with the cybervictimization experience. Informal support was more common; of 52 respondents, a total of 37 (71%) received support from their families. When asked about how helpful it was, family support received variable ratings as very good (14/37, 38%), good (10/37, 27%), and poor (11/37, 30%). Most victims also received support from their friends (40/52, 77%), which they rated as primarily very good (17/40, 42%).

Formal support was less common, and the number of respondents varied. It included approaching victim support groups (20/50, 40%), which were generally rated as poor (11/20, 55%). Health care professionals were also approached (22/52, 42%), and this was mainly rated as very good (10/22, 45%). When asked specifically about their general practitioners, only some participant (13/53, 25%) stated they have disclosed to their general practitioners about what they were going through. The police were contacted by victims (20/52, 38%) and were mainly rated as poor (13/20, 65%). The support sought by victims and the perceived effectiveness of the support are detailed in Table 7.

**Table 7:** Informal and formal support sought by the victims and the perceived effectiveness of the support provided.

Support channel	Yes (approached this channel), n (%)	Rating of support received, n (%)					No (did not approach), n (%)
		Poor	Fair	Good	Very good		
Family (n=52 total responses)	37 (71)	11 (21)	2 (4)	10 (19)	14 (27)	15 (29)	
Friends (n=52 total responses)	40 (77)	7 (13)	7 (13)	9 (17)	17 (33)	12 (23)	
Victim support groups (n=50 total responses)	20 (40)	11 (22)	4 (8)	3 (6)	2 (4)	30 (60)	
Health care professional (n=52 total responses)	22 (42)	6 (12)	4 (8)	2 (4)	10 (19)	30 (58)	
Police (n=53 total responses)	20 (38)	13 (25)	1 (2)	3 (6)	3 (6)	33 (62)	
Other channels (n=49 total responses)	18 (37)	6 (12)	2 (4)	4 (8)	6 (12)	31 (63)	

## Discussion

### Principal Findings

This cross-sectional study represents the quantitative phase of a mixed methods research to examine the scope of cybervictimization experiences among people with long-term conditions and disabilities in the United Kingdom and how it affected their self-managed health plan. Approximately 1 in every 2 people with long-term conditions in this study experienced cybervictimization. The sample was diverse in demographics, such as age and ethnic groups, with most participants (120/152, 78.9%) being female. The participants reported a range of chronic conditions and impairments that were grouped using the ICD-10. Most changes to the self-management plan were under the lifestyle category in addition to changes to medications, follow-up, and self-monitoring. The participants perceived lower self-efficacy, which potentially affected their self-management.

The most common means of contacting the victims was Facebook, and most harassers were strangers. Statistical tests were significant among cybervictimization and disability, fear or distress, the perceived impact of cybervictimization on health, and long duration of abuse (more than a year). Support was sought through formal and informal channels, with the former generally rated as poor.

### Comparison With Prior Work

It is challenging to compare the scope of cybervictimization among people with long-term conditions with the literature. This is mainly because the prevalence of cybervictimization depends on the definition and criteria adopted by the researchers to describe a negative web-based experience, which varies [11,25]. This remains an issue. A recent review [39] highlighted the challenges of prevalence inconsistencies in the cybervictimization literature because of issues with definitions and methodological variations in addition to contextual factors, including culture and geographical settings. Among people with chronic conditions, cybervictimization was reported to be as high as 41.7% [14]; however, this was in a younger age group and in a different context than that in this study. It is important to acknowledge cybervictimization as a global health issue, and further work is needed to tackle inconsistencies in definitions to have a clearer understanding and facilitate conversations among researchers internationally.

Most of the participants in this study (120/152, 78.9%) were female, with no statistically significant difference between the genders. In the current literature, studies that examine the cybervictimization phenomenon and its impact on different groups are inconsistent; in some cases, cybervictimization was associated with the male gender [40], and in other cases, it was associated with the female gender [41,42]. Notably, most studies that focused specifically on victimizing people with disabilities were male dominated [43-45], and some studies showed increased cybervictimization toward girls with disabilities [46]. This could be influenced by several factors, such as the young age group in previous studies or focusing on specific disabilities that are more common among male individuals, such as attention-deficit/hyperactivity disorder. Hence, this study added to the literature by reporting the experiences of people with long-term conditions with input from women. Further research is needed to examine whether this result reflects attitudes toward participation and higher cybervictimization among women or whether cultural factors have influenced the results, for example, if men are seen as masculinity figures who should not disclose similar experiences.

The participants in this study were all adults aged ≥18 years. This is an important addition to the literature. Previous studies on cybervictimization have focused on young age groups [11], and how cybervictimization affects older populations remains underresearched [39]. A review on behalf of the Department for Digital, Culture, Media, and Sport examined the evidence on the harms of web-based experiences on adults and acknowledged the scarcity of evidence in examining disability hate against adults [47].

Most victims in this study (53/69, 77%) had disabilities, and there was a statistically significant relationship between cybervictimization and disability. This is in line with previous research on cyberharassment and disability [48] as well as research examining cyberbullying among younger age groups [12,13,49]. In addition, almost half of the victims (22/53, 42%) considered victimization to be related to their condition or impairment. An explanation could be the targeting of people with physical impairments by harassers. This is in line with the role of disability discrimination and hate in the literature [48,50]. It is alarming to see disability discrimination taken to a web-based context, which can potentially lead to cyberincidents or crimes. This study focused on people with long-term conditions, and this significant association that builds on the existing literature makes disability and cybervictimization a research area to be examined by multidisciplinary teams.

The characteristics of the entire sample and those of the victims with long-term conditions were comparable. The age of the victims was slightly higher in those with disabilities. This finding is unlike the literature that focused on cyberbullying among children [43,45], showing how victimization continues throughout the life course. Employment status was lower and there were fewer professionals among victims, more so among victims with disabilities. This could be due to restricted physical activity in some physical or invisible impairments [16]. However, this could also reflect accessibility issues, marginalization, and stereotyping of people with disabilities [50]. Despite the slight differences, the sample, victims, and victims with disabilities had comparable characteristics, suggesting an alarming risk of being victimized across all groups.

Most victims in this study (60/68, 88%) experienced fear and distress, which is consistent with previous studies [25]. The relationship between fear and cybervictimization impact was statistically significant. This perceived impact was also significant in cases with longer durations, which extends the literature and could be used for awareness raising and health promotion to prevent long-term health consequences. Fear and eliciting distress were factors used in previous studies to examine the impact of cyberharassment [48], and eliciting distress was also included in defining cyberstalking [25]. Fear can also be viewed as a precursor to harm, which can be physical or mental. Although fear is reported here as an impact as it might influence how the individual manages the chronic condition and results in health consequences, it can also be viewed as a factor to build on for future interventions. For example, the fear of safety was one of the factors that facilitated the reporting of cyberhate cases to the police [51].

The diversity of reported conditions in this study ensured that we covered different impairments, scoping the impact on each condition and directing future research. In the literature, only a few of the conditions reported here are reported collectively, and none are specifically reported in relation to victimization [11]. Asthma was the most frequently reported condition in this study. The impact of victimization on managing asthma has been studied previously among young patients [13,52]; however, it has not been examined at a later age. Diabetes was highly prevalent in the sample, which could reflect its prevalence in the general population and documented victimization [13]. Patients with thyroid diseases were also victimized; however, this has not been studied before and requires further research. These findings do not exclude people with other conditions; rather, they warn of the increase in cybervictimization and the need for research to examine the specific impact on health conditions.

Anxiety and depression were also reported in the sample and were exacerbated by cybervictimization, which is concerning considering the distress caused by the experience itself [25]. Individuals with autism spectrum disorders and Asperger syndrome were included. However, the impact and victimization of people with these conditions were lower than expected compared with previous studies [44,45,53]. However, this comparison is not conclusive because of the low number of these participants. This could be influenced by the recruitment process and, thus, requires further research. Such findings reflect the wide range of conditions included; they might also suggest differences in impact compared with younger victims or could be a result of methodological differences.

Invisible conditions such as MS and ME were highly reported. The victimization of people with invisible disabilities has been documented [10] and was further confirmed in this study. Patients with epilepsy also shared the impact of cybervictimization on their self-management. Previous studies have shown that people with epilepsy are victimized offline [54] or on the web at a young age [13], confirming that people with conditions documented to be victimized offline but not studied on the web or among adults could be at risk of cybervictimization.

Diseases of the musculoskeletal system and connective tissue disorders were reported by the victims, and they require further research concerning cybervictimization. EDS is a rare condition in epidemiology [1]. Nonetheless, it was a considerable concern for the participants. The representation of invisible and less common conditions could be linked to the participants’ identities and attitudes toward participation [34].

In total, 61% (33/54) of victims reported that experiencing cybervictimization affected their self-management plans. Previous research has not specified changes in managing health after victimization [11,13,47,49]. After cybervictimization, the reported impact on self-management was mainly on avoiding triggers, healthy eating, and avoiding exercise. The importance of this lies in the specific characteristics of each condition. Lifestyle changes are broad, and triggers are different in each management plan [55]. In addition, healthy eating and exercise are essential aspects of self-management, for example, in diabetes, musculoskeletal conditions, and depression. Moreover, triggers of neurological, mental health, and heart conditions can have an immediate effect [56]. Regular medications were also affected. Missing medications, for example, in heart disease and diabetes, can trigger life-threatening situations [57]. This indicates the need to raise awareness to prevent such serious complications.

In this study, 69% (38/55) of victims perceived a worsened self-efficacy for the self-management of health conditions following cybervictimization [30]. It is acknowledged that such results do not quantify the impact of cybervictimization and that the participants already experienced fear. However, the results reflected the victims’ perceptions of how this experience affected their coping. Thus, it could be used as a rough estimate to demonstrate the health disruption caused by cybervictimization.

By examining the population at risk of cybervictimization, the diversity of the included conditions, and the multilevel impact on self-management, it can be argued that cybervictimization is a threat to public health. This is in line with previous work that acknowledged that cybervictimization results in unexpected health consequences and, in turn, health-associated costs to individuals and systems [39]. Identifying cybervictimization as a global health issue is an essential step in an increasingly connected world with massive web-based communication. During the COVID-19 pandemic, web-based experiences changed, cybervictimization risks increased [58], and more hate crimes were reported in the United Kingdom [59-61]. In public health emergencies and without proper action, people with long-term conditions may face long- and short-term health consequences.

### Strengths and Limitations

This study contributes to the body of literature by focusing on adults as an age group and addressing a diverse range of health conditions and impairments. The researchers aimed to give every person living with a chronic condition in the United Kingdom the opportunity to participate. However, equal chances for participants in this study were influenced by the recruitment strategy as gatekeepers were approached during recruitment. The researchers recognize the influence of the recruitment process on the results and do not claim the generalizability of the findings. However, the findings provide an idea of the frequency and interrelationship between having a chronic condition and cybervictimization experience and its impact on self-management. In addition, the recruitment was inclusive of participants facing physical barriers and people who were determined to share their voices, for example, disability rights advocates. The sample in this study was not large; however, the study was specifically designed to examine cybervictimization without treating chronic conditions as a homogeneous group. Previous studies have used existing data sets that were not specifically designed for this topic, and chronic conditions have been mostly reported in large samples as a homogeneous group [11]. Hence, the study design and specific conditions will guide future research.

Using a web-based approach to reach participants was an inclusive option given the range of health conditions and the sensitivity of the topic. However, this approach was also challenging. The challenges faced by this study during the recruitment stage have been published elsewhere [34]. The lack of internet access and socioeconomic status are also limiting factors to consider [62], as well as social desirability bias in self-reporting [63]. This was managed by designing the survey in such a way that more than one question was assigned to address 1 issue; for example, 2 questions covered cybervictimization experience, and 4 questions covered the impact on self-management. In addition, we encouraged the participants to elaborate on their experiences in the qualitative phase of the study.

The self-efficacy scale used was validated. However, the participants were asked about their self-efficacy before and after cybervictimization at a single point during data collection. Hence, the scores are not conclusive, and they might be influenced by recall bias or exaggerated in cases of ongoing harassment or mental health impact. This question was used to examine perceived impact in combination with other questions on the impact of cybervictimization rather than as a stand-alone score.

### Conclusions and Future Directions

This study pioneered research on cybervictimization of people with long-term conditions in the United Kingdom and identified the need to build proper support that is context and condition specific. Reaching context-specific work could be refined in future research, and a health condition–specific work can be achieved by using these findings to identify possible conditions that were targeted and their potential impact, which could help tailor specific prevention interventions and support by experts in the field. All the conditions reported in this study require attention and further investigation because of their potential impact on victims. It is also essential to tackle inconsistencies in definitions and recognize cybervictimization as a global health issue that requires international conversations and consistent language to grasp the scope of the issue and potential interventions. Further research is also needed to examine how public health emergencies in the age of web-based communication, such as the COVID-19 pandemic, have influenced the web-based experiences and health outcomes of people with long-term conditions and disabilities. The victimization of people with chronic health conditions, especially those with disabilities, will continue if we do not take a holistic approach to tackling this pressing issue.

## Abbreviations

EDS: Ehlers-Danlos syndrome
ICD-10: International Classification of Diseases, 10th Revision
ME: myalgic encephalomyelitis
MS: multiple sclerosis

## Data Availability

The data set was not placed in an open repository owing to the sensitivity of the topic.
